# Polyelectrolyte Chondroitin Sulfate Microgels as a Carrier Material for Rosmarinic Acid and Their Antioxidant Ability

**DOI:** 10.3390/polym14204324

**Published:** 2022-10-14

**Authors:** Mehtap Sahiner, Selin S. Suner, Aynur S. Yilmaz, Nurettin Sahiner

**Affiliations:** 1 Bioengineering Department, Engineering Faculty, Canakkale Onsekiz Mart University Terzioglu Campus, Canakkale 17100, Turkey; 2 Department of Chemistry & Nanoscience and Technology Research and Application Center, Canakkale Onsekiz Mart University Terzioglu Campus, Canakkale 17100, Turkey; 3 Department of Ophthalmology, Morsani College of Medicine, University of South Florida, Tampa, FL 33612, USA; 4 Department of Chemical and Biomolecular Engineering, University of South Florida, Tampa, FL 33620, USA

**Keywords:** chondroitin sulfate (CS), polyelectrolyte microgels, biocompatible, rosmarinic acid, antioxidant

## Abstract

Polyelectrolyte microgels derived from natural sources such as chondroitin sulfate (CS) possess considerable interest as therapeutic carriers because of their ionic nature and controllable degradation capability in line with the extent of the used crosslinker for long-term drug delivery applications. In this study, chemically crosslinked CS microgels were synthesized in a single step and treated with an ammonia solution to attain polyelectrolyte CS^−^[NH_4_]^+^ microgels via a cation exchange reaction. The spherical and non-porous CS microgels were injectable and in the size range of a few hundred nanometers to tens of micrometers. The average size distribution of the CS microgels and their polyelectrolyte forms were not significantly affected by medium pH. It was determined that the −34 ± 4 mV zeta potential of the CS microgels was changed to −23 ± 3 mV for CS^−^ [NH_4_]^+^ microgels with pH 7 medium. No important toxicity was determined on L929 fibroblast cells, with 76 ± 1% viability in the presence of 1000 μg/mL concentration of CS^−^[NH_4_]^+^ microgels. Furthermore, these microgels were used as a drug carrier material for rosmarinic acid (RA) active agent. The RA-loading capacity was about 2.5-fold increased for CS^−^[R]^+^ microgels with 32.4 ± 5.1 μg/mg RA loading, and 23% of the loaded RA was sustainably release for a long-term period within 150 h in comparison to CS microgels. Moreover, RA-loaded CS^−^[R]^+^ microgels exhibited great antioxidant activity, with 0.45 ± 0.02 μmol/g Trolox equivalent antioxidant capacity in comparison to no antioxidant properties for bare CS particles.

## 1. Introduction

Biological polyelectrolytes (PECs), also called biological polyelectrolyte complexes, generally comprise strong intermolecular interactions known as Coulomb’s interactions or electrostatic interactions between oppositely charged groups, e.g., cationic biopolymers such as chitosan [1], poly-l-lysine [2], poly-L-arginine [3], insulin [4], collagen [5], amino dextran, and 2-(diethylamino) ethyl dextran [6] with anionic biopolymers such as pectin [7], alginate [8], xanthan gum [1], dextran derivatives [6], hyaluronic acid [9], carrageenan [1], neem gum [10], heparin [11], chondroitin sulfate [12], carboxymethyl cellulose [13], humic substances [14], poly(γ-glutamic acid) [15], DNA [16], and siRNA [17]. Surfactant systems are generally used to prepare different polymeric systems, including spherically shaped polymeric particles at different sizes. PECs can be formed without the need for chemical-crosslinking agents or surfactants, making their synthesis much easier [12]. Apart from this, some anionic biopolymers can readily interact with three valent metal ions, e.g., Gd(III) and Fe(III), to prepare biological polyelectrolyte complexes [9]. Some parameters, including molecular weights of the complex forming polymers [12], charge-to-charge stoichiometry, and charge density of anions and cations, as well as pH, ionic strength, and concentration of the reaction medium, have effects on the formation and stability of the resultant polyelectrolytes [17].

PEC assemblies have been used in wide range of applications, such as tissue adhesives and scaffolds for tissue engineering [18], hematostats [19], biosensors [19], implantable materials [1,17,20], templates for enzyme immobilization [21], antimicrobial agents [22], and especially, drug delivery devices [1]. The interaction between the opposite components of PECs also ensures high adherence to biological tissues and, thus, renders mucoadhesive biological activity [21,23]. PEC-based biomaterials, such as a chitosan-chondroitin sulphate complex, could exhibit specialized properties for chronic wound healing, providing higher cell density [12]. Amphiphilic structures of PECs provide various advantages in drug reservoirs, such as improved encapsulation efficacy of drugs by strong intra- and inter-chain interactions and the sustainable release of drugs in mucous membranes [1,17,20]. Furthermore, PECs can exhibit antibacterial activities depending on their polyanionic or polycationic contents and their nature. In a study, a magnesium alloy covered with lysozyme as polyanionic content and polyethyleneimine as polycationic content was used to create polyelectrolyte films, and these films showed great antibacterial activity, as well as antiplatelet adhesion related to the antimicrobial nature of the ionic compounds [24].

Chondroitin sulfate (CS) is an anionic natural polymer that consists of anionic functional sulfate and carboxylic acid groups [23,25]. CS is also known to be found in mammalian tissue attached to proteoglycans in the extracellular matrix (ECM) and, thus, possess properties such as non-toxicity, biocompatibility, and biodegradability [20,26]. CS can take part in the scaffolding of materials for biomedical purposes due to its many biobeneficial properties, such as anti-inflammatory, antioxidant, antineoplastic, and anticoagulant activities [27]. In addition to these, CS-based coating supports the repairing process of skin and cartilage tissues [12,20]. Due to its glycosaminoglycan structure, CS-based coatings have shown growth-factor-binding ability and can be used to stabilize and deliver growth factors, which regulate wound-healing activities [25,26,27]. These valuable properties of CS make its different formulations valuable materials for pharmaceutical and biomedical applications as significant biomolecules in the design of polyelectrolyte complexes. It has been reported that CS as an anionic biopolymer interacts with chitosan [12], peptides [28], pectin [29], and cationic tannin [18] for various biomedical purpose. For example, CS-based polyelectrolytes have been prepared through the interactions of CS polymer chains with metal ions, such as Gd(III) ions as a magnetic resonance imaging (MRI) enhancer [30], with Al(III) ions as a polyphenolic carrier [31], and with Zn(II) ions to provide antibacterial ability [32].

In this study, a chondroitin sulfate (CS) biopolymer is chemically crosslinked using divinyl sulfone (DVS) as a crosslinker in a reverse micelle microemulsion system to prepare CS microgels, which can also be called polymeric particles, in the size range of nanometers to micrometers. Degradable and non-degradable CS microgels can be prepared by changing the DVS crosslinker ratio in the polymeric network, as reported by our previously study [33]. CS microgels afford great potential as antibiotic carrier biomaterials for bacterial infections because of their tunable degradability, good biocompatibility, and especially, long-term drug delivery capability for antibacterial activities. The goal of this study is to prepare polyelectrolyte CS^−^[R]^+^ microgels through the treatment of CS microgels with ammonia in an aqueous solution to obtain a microgel system for sustainable and long-term therapeutic release capability. Most PECs are not stable at broad pH ranges because of the weak physical interactions between anion and cation moieties that are present in the PEC networks. As carrier materials, PECs often show burst drug release ability in delivery applications due to complete degradation at suitable pH conditions [34]. PEC hydrogels that can be chemically crosslinked with amphoteric materials have recently attracted much attention as active agent delivery applications [35]. Therefore, CS^−^[R]^+^ microgels as a PEC structure can possess high stability in physiological solutions and offer great potential for higher drug-loading capacities and longer delivery times for certain types of drugs. The pH responsiveness, size distribution, and zeta potential values of CS microgels and CS^−^[R]^+^ microgels are also determined in the pH range from 2 to 11. The cytotoxicity of CS and CS^−^[R]^+^ on L929 fibroblasts for 24 h of incubation time, as well as their Fe(II)-chelating percentages, were investigated. Furthermore, rosmarinic acid (RA) as a therapeutic agent, a well-known antioxidant and anti-inflammatory agent [36], is loaded onto CS microgels and CS^−^[R]^+^ microgels, and their RA release profiles are investigated at physiological conditions, in pH 7.4 phosphate-buffered solution (PBS), and at 37 °C. The antioxidant abilities of bare and RA-loaded CS and CS^−^[R]^+^ microgels are also investigated using total phenol content (TPC) and ABTS scavenging assays and compared with RA.

## 2. Materials and Methods

### 2.1. Materials

In the synthesis of the CS microgels, chondroitin sulfate A sodium salt (CS, ≥98%; average MW, 10,000–30,000; Biosynth carbosynth, San Diego, CA, USA) as a linear polymer, divinyl sulfone (DVS, 97%; Merck, Darmstadt, Germany) as a crosslinker, dioctyl sulfosuccinate sodium salt (AOT, 96%; Acros Organics, Geel, Belgium) as a surfactant for the emulsion medium, and 2,4-trimethylpentane (isooctane, ≥99.5%; Isolab, Laborgeräte GmbH, Eschau, Germany) as a solvent for the emulsion medium were used as received. An ammonia solution (25%; Sigma-Aldrich, Milwaukee, WI, USA) was used to prepare of polyelectrolyte forms of CS microgels. Potassium nitrate (KNO_3_; granular, Fisher Chemical, Waltham, MA, USA) was used for zeta potential and size distribution measurements. For the cytotoxicity analysis, L929 fibroblast cells (mouse C3/An connective tissue; SAP Institute, Ankara, Turkey) were used. In the cell culture studies, trypsin (0.25%; EDTA 0.02% in PBS), Dulbecco’s Modified Eagle’s Medium (DMEM with 4.5 g/L glucose, 3.7 g/L sodium pyruvate, and 0.5 g/mL L-Glutamine), fetal bovine serum (FBS, heat inactivated), and penicillin/streptomycin (10,000 U/mL penicillin and 10 mg/mL streptomycin) were obtained from Panbiotech. As an MTT agent, 3-(4,5-dimethylthiazol-2-yl)-2,5-diphenyltetrazolium bromide (98%) was purchased from neoFroxx GmbH (Hesse, Germany). Fe(II) sulfate heptahydrate (FeSO_4_ 7H_2_O; Merck; 99.5%) and 5,6-diphenyl-3-(2-pyridyl)-1,2,4-triazine-4,4-disulfonic acid disodium salt hydrate (Ferrosine; Alfa Aesar; 99%) were used for the iron(II) chelation assay. Rosmarinic acid (RA, 96%; Aldrich, USA) was used as an active agent for the drug-loading study. Folin–Ciocalteau’s phenol reagent (FC; Sigma-Aldrich, USA), gallic acid (GA, 97.5–102.5%; Sigma-Aldrich, USA), potassium persulfate (KPS, 99%; Sigma-Aldrich, USA), 2,2′-azino-bis-(3-ethylbenzothioazoline-6-sulfonic acid) (ABTS, >98%; HPLC-grade; Sigma-Aldrich, USA), and (±)-6-hydroxy-2,5,7,8-tetramethylchromane-2-carboxylic acid (Trolox, 97%; Aldrich, USA) were used for antioxidant studies. As solvents, acetone (99%; Birkim, Istanbul, Turkey), dimethyl sulfoxide (DMSO, 99.9%; Carlo Erba, Val-de-Reuil, France), and ultra-pure deionized water (18.2 M.Ω. cm resistivity; Millipore Direct-Q 3 UV water purification system) were used as received.

### 2.2. Synthesis of CS Microgels

The synthesis of CS microgels was carried out via the reverse micelle microemulsion polymerization technique described by Suner et al. 2022 [33]. Briefly, 0.3 g of CS was dissolved in 10 mL of 0.2 M NaOH solution and 1 mL of the prepared CS solution was dispersed into 30 mL of 0.2 M AOT/isooctane solution. After that, as a crosslinker, DVS at a 50% mole ratio relative to a repeating unit of CS was added into the solution. The resulting emulsion medium was rapidly vortexed and stirred for 2 h at a 1000 rpm mixing rate at room temperature to obtain crosslinked CS microgels. After 2 h, CS microgels were precipitated into excess acetone and kept overnight. The supernatant was carefully decanted, and the remaining microgels were washed with an acetone:water (50:50, *v*:*v*) mixture once and with acetone twice to remove unreacted chemicals and surfactants by centrifugation at 10,000 rpm for 10 min. The washed CS microgels were dried with a heat gun and kept for further use.

### 2.3. Preparation of Polyelectrolyte CS^−^[R]^+^ Microgels

Polyelectrolyte CS^−^[R]^+^ microgels were attained through a cation exchange reaction with an ammonium hydroxide treatment of CS microgels. Shortly, 0.2 g of CS microgels were suspended in 50 mL of 6.25% aqueous ammonia solution and stirred at 200 rpm for an hour. The polyelectrolyte CS^−^[R]^+^ microgels were precipitated by centrifugation at 10,000 rpm for 10 min and washed with an acetone:water (50:50, *v*:*v*) mixture once and acetone one time. The obtained CS^−^[R]^+^ microgels were dried with a heat gun and kept for further use.

### 2.4. Characterization of CS Microgels and Polyelectrolyte CS^−^[R]^+^ Microgels

Morphological analysis of the CS microgels was performed with a scanning electron microscope (SEM; SU70, Hitachi, Japan). Briefly, CS microgels were covered with palladium and gold to a few μm under a vacuum for 10 s and visualized at 10.0 kV. The swollen ability of CS microgels and CS^−^[R]^+^ microgels at different pH levels ranging between 2 and 11 were analyzed using dynamic light-scattering (DLS; Brookhaven Nanobrook Omni, Holtsville, NY, USA) measurements. In brief, 1 mg/mL concentration of CS-based microgels was suspended in 1 mM of KNO_3_ aqueous solution and filtered with a 5 μm pore size syringe filter for hydrodynamic average size distribution analysis. Furthermore, zeta potential was determined using 40 mg of CS-based microgel suspension in 40 mL of 1 mM KNO_3_ solution, and the zeta potential was measured against pH using a zeta-potential-measuring device (Brookhaven Nanobrook Omni, Holtsville, NY, USA). DLS and zeta potential analyses were repeated ten times, and the results were given with standard deviations. Fourier-transform infrared (FT-IR) spectra of the CS-based microgels were recorded in the frequency range of 4000 to 650 cm^−1^ with 4 cm^−1^ resolutions using an FT-IR spectrophotometer (Perkin-Elmer, Spectrum 100, Akron, OH, USA). Thermal gravimetric analysis (TGA; Seiko, SII TG/DTA 6300, Tokyo, Japan) of 5 mg of CS-based microgels was examined from a 50 to 700 °C temperature range at a heating rate of 10 °C/min under a 100 mL/min nitrogen flow rate.

### 2.5. Biocompatibility of CS Microgels and Polyelectrolyte CS^−^[R]^+^ Microgels

#### 2.5.1. Cell Culture

Cytotoxicity analyses of CS microgels and CS^−^[R]^+^ microgels were performed for L929 fibroblast cells via MTT assay according to the procedure described by Suner et al. [33]. L929 fibroblast cells (mouse C3/An connective tissue) were used in the cytotoxicity analysis. The fibroblast cells were cultured in DMEM supplemented with 10% FBS and 1% penicillin/streptomycin antibiotics using a 25 cm^2^ flask at 37 °C in a 5% CO_2_:95% air atmosphere for 24 h.

#### 2.5.2. Cytotoxicity of CS Microgels and Polyelectrolyte CS^−^[R]^+^ Microgels

CS microgels and CS^−^[R]^+^ microgels were suspended in DMEM in order to obtain an initial concentration of 1000 μg/mL. This sample was adjusted to 500, 250, 100, and 50 μg/mL concentrations by diluting with DMEM solution. The stock L929 fibroblast cell cultures were seeded in 96-well plates with approximately 1 × 10^5^ cells for each well in 0.1 mL of DMEM, and the plates were incubated in a 5% CO_2_:95% air atmosphere at 37 °C for 24 h. After the incubation time, the media in the well were removed, and various concentrations from 1000 μg/mL to 50 μg/mL particle suspensions in 100 μL DMEM were added on the attached cells in the wells and incubated for 24 h. For positive control, only 100 μL DMEM was added in the wells. Following the incubation period, the culture media were removed, and cells were washed with phosphate-buffered solution (PBS). Then, 5 mg/mL of MTT agent was diluted 10-fold in DMEM, and 100 μL of this agent solution was added to each well. The 96-well plates were kept in the dark for 2 h. Finally, the media were discarded, and 200 μL of DMSO was added to each well to dissolve of the formazan crystals. Absorbance values at 590 nm were read using a microplate reader (HEALES, MB-530). The analysis was repeated three times, and the values were reported as the average values with standard deviations.

### 2.6. Fe(II)-Chelating Capacity of CS Microgels and Polyelectrolyte CS^−^[R]^+^ Microgels

In accordance with the literature, CS microgel and CS^−^[R]^+^ microgel aqueous solutions were prepared at 2000 µg/mL concentrations and were diluted to 1000, 500, 250, and 125 µg/mL. Then, 140 µL of CS microgel or CS^−^[R]^+^ microgel suspension solution was added into 96 wells [37]. Then, 20 µL of 1 mM Fe (II) aqueous solution was added to them, and measurements were obtained using a microplate reader (Thermo Multiskan Go, USA) at 562 nm. Finally, 40 µL of 2.5 mM ferrozine aqueous solution was added, and the measurements were performed again. Pure water was used for control. The results were calculated according to the literature.

### 2.7. Rosmarinic Acid (RA) Loading and In Vitro RA Release from CS Microgels and CS^−^[R]^+^ Microgels

Rosmarinic acid (RA) as a therapeutic agent was loaded into CS microgels and CS^−^[R]^+^ microgels through an adsorption technique. Briefly, 0.03 g of RA was dissolved in 30 mL of a 1:1 volume ratio of ethanol:water solution, and 0.15 g of CS-based microgels was added into an RA-solution-containing container. For the loading process, the microgel suspension was stirred at 300 rpm for 6 h. After this period, the microgels were washed with a 1:1 volume of ethanol:water mixture by centrifugation at 10,000 rpm for 10 min and dried by freeze-drying. The RA-loading amounts were determined from the absorbance of the drug solution before and after the loading process using UV-vis spectroscopy (T80+ PG Instrument) at 325 nm against the previously created corresponding calibration curves of RA prepared in ethanol:water mixture.

In the drug release study, 50 mg of RA-loaded CS microgels or CS^−^[R]^+^ microgels was dispersed in 1 mL of phosphate-buffered saline (PBS) at pH 7.4 and transferred to a dialysis membrane. This particle-containing membrane was placed into 20 mL of PBS solution at 37 °C in a shaker bath. The drug-releasing medium, the PBS solution, was then sampled and evaluated with a UV-vis spectrometer (T80+ PG Instrument) at 325 nm to measure the amount of RA against the previously determined corresponding RA calibration curves prepared in PBS, and the released amounts of RA were calculated. The analysis was repeated three times, and the values were reported as the average values with standard deviations.

### 2.8. Antioxidant Properties of Bare and RA-Loaded CS and CS^−^[R]^+^ Microgels

#### 2.8.1. Total Phenol Content of Bare and RA-Loaded CS and CS^−^[R]^+^ Microgels

The total phenolic contents of the particles were evaluated using the Folin–Ciocalteau (FC) method according to the literature with some modifications [38]. Briefly, 0.1 mL of 1 mg/mL microgel suspension was reacted with 1.25 mL of 0.2 N solution of FC phenol reagent for 4 min. Next, 1 mL of 0.7 M sodium bicarbonate solution was added to this mixture and kept in the dark for 2 h. Then, the total phenol content of the microgels was measured using a UV-vis spectrophotometer (T80+ PG Instrument) at 760 nm. The antioxidant activity of the microgels was expressed as μg/mL gallic acid equivalent. The analysis was repeated three times, and the values were reported as the average values with standard deviations.

#### 2.8.2. Antioxidant Properties of Bare and RA-Loaded CS and CS^−^[R]^+^ Microgels by ABTS Scavenging Assay

The antioxidant capacities of bare and RA-loaded CS microgels and CS^−^[R]^+^ microgels and RA were evaluated with an ABTS scavenging assay in accordance with the literature with slight modification [38]. An ABTS radical solution was prepared by mixing 2.5 mL of 2.45 mM potassium persulfate and 7.5 mL of 7 mM ABTS solution in DI water; the mixture was kept in the dark for 12 h at 4 °C to obtain a stock ABTS^•+^ solution. The stock ABTS^•+^ solution was diluted with PBS to adjust to an absorbance of 0.7 ± 0.05 at 734 nm using a UV-vis spectrophotometer (T80+ PG Instrument). Then, a 1 mg/mL concentration of each microgel suspension was prepared in 5 mL of PBS solution, and various amounts of this suspension from 200 to 500 µL were reacted with 3000 µL of ABTS^•+^ solution for 6 min. Separately, a 0.1 mg/mL concentration of RA solution in PBS was prepared, and 25–75 μL of this solution was interacted with 3000 µL of ABTS^•+^ solution for 6 min. At the end of this time, the decrease in absorbance value was detected at 734 nm. The antioxidant materials were determined for the values of 20−80% reduction of the blank absorbance. Trolox equivalent antioxidant capacity (TEAC) values were determined against the slope of a Trolox standard curve and expressed as “μmol Trolox equivalent/g”. The analysis was repeated three times, and the values were reported as the average values with standard deviations.

#### 2.8.3. Antioxidant Properties of Bare and RA-Loaded CS and CS^−^[R]^+^ Microgels by DPPH Assay

A 2,2-Diphenyl-1-picrylhydrazy (DPPH) assay was performed following the previously reported protocol described by Panda et al., with some modifications [39,40]. Briefly, DPPH solution was prepared in ethanol at a 100 µM concentration. Then, 10 mg of CS, CS^−^[R]^+^, RA-loaded CS^−^ or RA-loaded CS^−^[R]^+^ microgels was added to 3 mL of DPPH solution and incubated for 1 h in a dark environment. The absorbance of the DPPH solution was measured at 517 nm. The radical scavenging activity percentage was determined as a decrease in the absorbance of DPPH by Equation (1). A_sample_ is the absorbance of the sample, and A_control_ is the absorbance of the blank (without sample):

DPPH radical scavenging activity % = ((A_control_ − A_sample_)/A_control_ ) × 100 (1)

## 3. Results and Discussion

**Figure 1:**
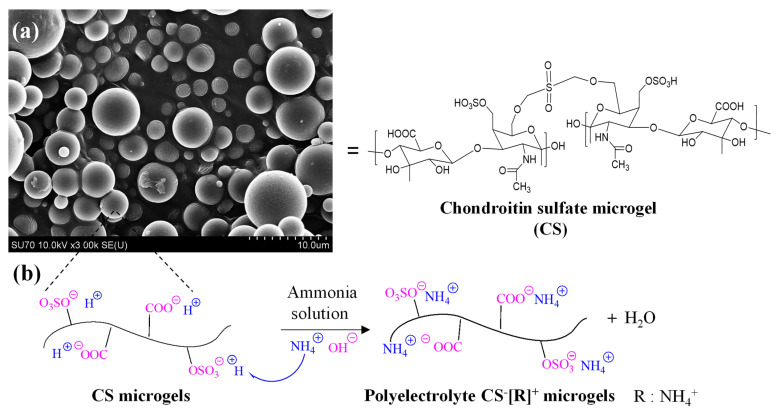
(**a**) SEM images of chondroitin sulfate (CS) microgels and (**b**) schematic representation of preparation of polyelectrolyte CS^−^[R]^+^ microgels via treatment with ammonia solution. [R = NH_4_^+^].

PEC-based hydrogels have great advantages as therapeutic delivery vehicles due to their higher affinities against cargo molecules with their amphiphilic groups and a more stable structure that can be attained by their crosslinked networks for a wide range of pH conditions [35]. In this study, polyelectrolyte CS^−^[NH_4_]^+^ microgels as biopolymeric PEC hydrogels were designed to carry rosmarinic acid for its sustainable release kinetics. CS^−^[NH_4_]^+^ microgels were prepared in two-step reactions, including the crosslinking of linear CS to generate CS microgels and ammonia treatment of the microgels to obtain polyelectrolyte forms of the microgels, or CS^−^[R]^+^ microgels. In the first step, non-degradable CS microgels were prepared employing a DVS crosslinker at a 50% mole ratio of a CS repeating unit, as reported previously [33]. Morphological analyses of the CS microgels were conducted through SEM imaging, as illustrated in Figure 1a.

The SEM images clearly show that the CS microgels were spherical, non-porous, and about 5 μm to submicron size ranges. To prepare polyelectrolyte forms of CS microgels, -OSO_3_^−^H^+^ or -COO^−^H^+^ groups of the CS microgels were treated with ammonia solution for 2 h at room temperature. As can be seen in the schematic representation of this treatment in Figure 1b, a cation exchange reaction was employed between H^+^ cations of the -OSO_3_^−^H^+^ and -COO^−^H^+^ groups with NH_4_^+^ cations from the ammonia solution [41]. Consequently, biological polyelectrolyte CS^−^[NH_4_]^+^ microgels could be prepared, which indicated that polyelectrolyte CS^−^[R]^+^ microgels could be made in a single step using ammonia solution related to anionic sulfonate groups of the CS microgels.

**Figure 2:**
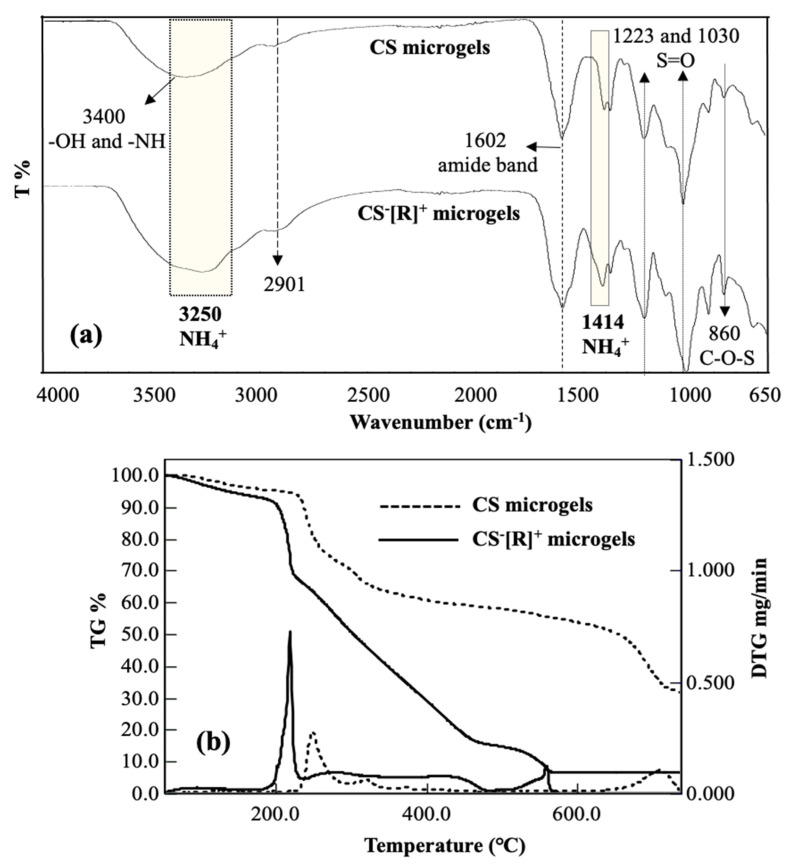
(**a**) FT-IR spectra and (**b**) thermogravimetric/differential thermogravimetric (TG/DTG) analysis curves of CS microgels and polyelectrolyte CS^−^[R]^+^ microgels.

The chemical structures of CS microgels and CS^−^[R]^+^ microgels were investigated by an FT-IR analysis that corroborated the crosslinking CS chains into CS microgels and the polyelectrolyte structure of CS^−^[NH_4_]^+^ microgels upon cation exchange, as given in Figure 2a. A broad peak was observed in the range of 3600–3000 cm^−1^ due to the O-H and N-H stretching of CS chains. The small peak at 2901 cm^−1^ was attributed to C-H stretching of the CH_2_ groups of CS. The sharp peak at 1602 cm^−1^ was assigned to the presence of an amide band in the CS structure. Additionally, the peak at 860 cm^−1^ corresponded to C-O-S groups of the CS structure in the microgel network. Among these CS peaks, the characteristic peaks of DVS were obtained at 1223, and 1030 cm^−1^ stretching vibrations belonged to S=O groups to confirm of the crosslinker structure of the CS microgels. The different peaks of polyelectrolyte CS^−^[R]^+^ microgels were determined at 3250 cm^−1^ by a broad band, and a 1414 cm^−1^ stretching vibration was attributed to NH_4_^+^ groups into the polyelectrolyte structure. These results supported that polyelectrolyte CS^−^[R]^+^ microgels were successfully prepared by the ammonia treatment of CS microgels.

The thermal degradation of CS microgels and CS^−^[R]^+^ microgels was acquired by a thermogravimetric/differential thermogravimetric (TG/DTG) analysis, and the corresponding TG/DTG curves are given in Figure 2b. The first slight degradation was shown in both thermograms at about 5.0% and 8.8% weight loss values at ~100 °C with slight DTG peaks corresponding to the loss of bound water from the microgel structures of CS and CS^−^[R]^+^ microgels, respectively. In addition, three main degradation steps occurred in the temperature ranges of 214–260 °C with a maximum peak at 250 °C and 23.1% weight loss; 260–400 °C with a maximum peak at 318 °C and 39.4% weight loss; and 620–738 °C with a maximum peak at 714 °C and 69.2% weight loss observed for CS microgels. The thermogram of the CS^−^[R]^+^ microgels showed three step degradations, and the first degradation was detected at a slightly lower temperature range of 190–230 °C, with a sharp DTG peak at 218 °C and 33.0% weight loss. More degradations were observed at the second degradation interval between 230–445 °C with two DTG peaks at ~282 and 422 °C with a total of 80.6% weight loss, and the third degradation step in the 450–560 °C range with a maximum peak at 560 °C and 84.8% weight loss. It was clearly seen that polyelectrolyte CS^−^[R]^+^ microgels were thermally more degradable than the CS microgels due to the presence of the ionic groups within the polymeric networks.

**Figure 3:**
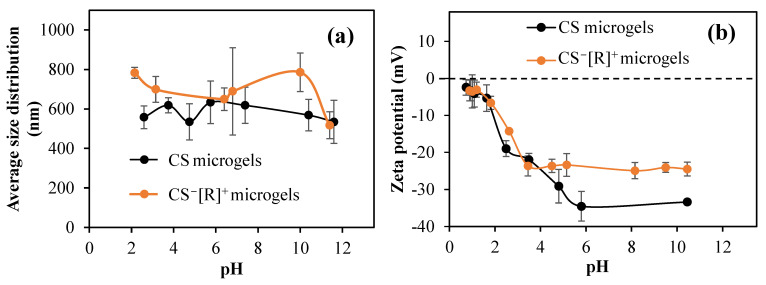
(**a**) Sizes of CS microgels and CS^−^[R]^+^ microgels at different pH values; (**b**) pH versus zeta potential graphics of CS microgels and CS^−^[R]^+^ microgels in pH 1–10 range.

Polyelectrolyte complexes and their microgels are stimuli-responsive materials against the pH and ionic strength of solvents and change their sizes and zeta potential values accordingly. Therefore, size measurements of the CS microgels and CS^−^[R]^+^ microgels at different solution pH levels were carried out with DLS studies. According to Figure 3a, the dimensions of CS microgels did not change much at different solution pH levels. The microgel sizes were varied between 534 and 633 nm. The size of the CS microgels was measured at 633 ± 104 nm at 5.7 pH. The size of the CS^−^[R]^+^ microgels also did not change significantly. As highly crosslinked (50%) particles were used as a tighter network of CS chains were constructed, the water swelling and ion–ion charge interactions were suppressed.

The zeta potentials of CS microgels and CS^−^[R]^+^ microgels in 1 mM KNO_3_ versus pH were measured. As presented in Figure 3b, the isoelectric point could not be detected in both microgels. The pH value measured in the 1 mM KNO_3_ solution for the CS microgel was 10.45, and the zeta potential at that pH value was −33.4 ± 0.7 mV. For the CS^−^[R]^+^ microgels, the measured pH was 8.5, and its zeta potential was −24.5 ± 2.1 mV.

**Figure 4:**
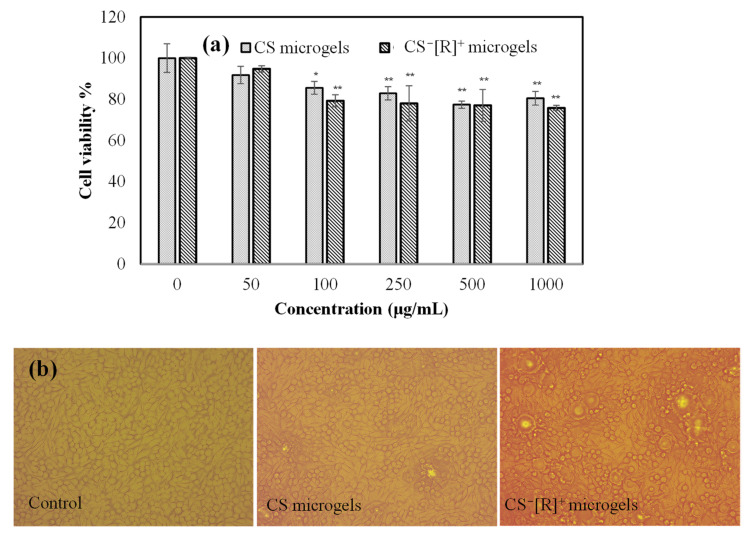
(**a**) Cell viability of L929 fibroblasts in the presence of different concentrations of CS and CS^−^[R]^+^ microgels and (**b**) optic microscope images of L929 fibroblast cells for control groups and 1000 μg/mL concentrations of CS microgels and CS^−^[R]^+^ microgels [* *p* < 0.005, ** *p* < 0.01 vs. control].

To ensure the safety of the biomaterials, a biocompatibility test, e.g., the effects of materials on the viability of healthy cells, such as L929 fibroblasts, needed to be studied. Therefore, the cytotoxicity levels of CS microgels and polyelectrolyte CS^−^[R]^+^ microgels at different concentrations ranging from 50 to 1000 μg/mL were determined on L929 fibroblast cells for a 24 h incubation time, and the corresponding results are demonstrated in Figure 4a.

Cell viability percentages of the fibroblasts in the presence of CS microgels and polyelectrolyte CS^−^[R]^+^ microgels, even at a high concentration, i.e., 1000 μg/mL, were found as 81 ± 5% and 76 ± 1%, respectively. It is clear that the cell viabilities of both CS-based microgels were almost similar and not significantly decreased up to a 1000 μg/mL concentration. Moreover, the cell image of the control group, which was non-treated cells, and the cell images incubated with 1000 μg/mL concentrations of CS microgels or CS^−^[R]^+^ microgels for 24 h are demonstrated in Figure 4b. It can be clearly seen that the cells interacting with both types of CS-based microgels were healthy and revealed almost similar cell viabilities with the control group. Therefore, it could be said that polyelectrolyte CS^−^[R]^+^ microgels were biocompatible against fibroblast cells and could be safely used for further in vivo applications at <1000 μg/mL concentrations.

**Figure 5:**
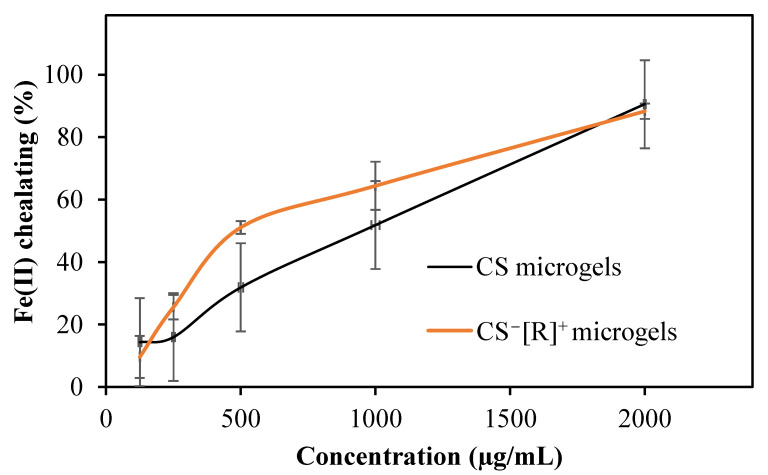
Fe(II)-chelating activities of CS microgels and polyelectrolyte CS^−^[R]^+^ microgels.

In the treatment of neurodegenerative diseases, such as Alzheimer’s and Parkinson’s, iron chelators are generally used as a therapeutic agent. Some polymers provide an iron-chelating ability and can be used to inhibit iron toxication in the body [38]. The Fe(II)-chelating capacities of CS microgels and CS^−^[R]^+^ microgels were examined in a DI water solution in the 125–2000 µg/mL concentration range in accordance with the literature [37]. In our previous work, the Fe(II) chelation ability of CS was not observed, even at a 2000 μg/mL concentration. However, as shown as Figure 5, it was observed that the Fe(II)-chelating capacity of CS microgels and CS^−^[R]^+^ microgels increased depending on the concentration.

As shown in Figure 5, CS had a Fe(II)-chelating capacity of 51.8 ± 15.8%, while that of CS^−^[R]^+^ microgels was increased to 64.5 ± 7.7% at the concentration of 1000 µg/mL due to the high-electrolyte nature of the microgels.

**Figure 6:**
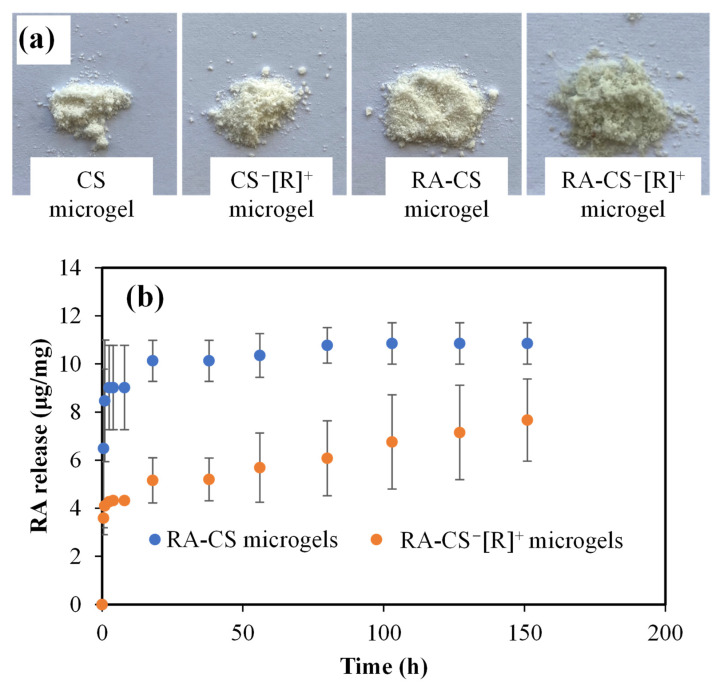
(**a**) Digital camera images of CS microgel and CS^−^[R]^+^ microgel and their RA-loaded forms. (**b**) RA release profiles of CS and polyelectrolyte CS^−^[R]^+^ microgels at physiological conditions, pH 7.4 (PBS), and 37 °C.

Rosmarinic acid (RA) is a well-known active agent due to its antioxidant, anti-inflammatory, antimutagenic, antimetastatic, antiangiogenic, neuroprotective, antibacterial, and antifungal activities and is widely recommended as a supplement in the human diet for its beneficial effects for various diseases [36]. The prepared CS microgels and polyelectrolyte CS^−^[R]^+^ microgels were used as RA carrier systems to attain a sustainable RA release and accomplish long-term therapeutic activity. Digital camera images of bare and RA-loaded CS microgels and polyelectrolyte CS^−^[R]^+^ microgels are demonstrated in Figure 6a.

As can be seen from the digital camera images given in Figure 6a, CS and CS^−^[R]^+^ microgels were white-colored, whereas RA-CS^−^[R]^+^ microgels were light-green-colored. Additionally, the color of the CS microgels did not change discernably upon RA loading into CS, as RA-CS microgels were white-colored. The RA-loading capacity of the CS microgels was measured as 13.5 ± 1.2 μg/mg RA, but almost 2.5-fold of the RA could be loaded into CS^−^[R]^+^ microgels, with a 32.4 ± 5.1 μg/mg RA-loading amount. It could be said that higher amounts RA could be loaded into CS^−^[R]^+^ as RA-CS^−^[R]^+^ microgels, and this provided a slight color change, with light green coming from the RA within the microgel network.

The RA release profiles from RA-CS microgels and RA-CS^−^[R]^+^ microgels at physiological conditions, pH 7.4 (PBS), and 37 °C are illustrated in Figure 6b. It is obvious that RA-CS microgels exhibited burst RA delivery within 20 h, with 10.8 ± 0.8 μg/mg RA delivery, whereas 7.6 ± 1.7 μg/mg RA was linearly released from RA-CS^−^[R]^+^ microgels. The RA release capacity of the polyelectrolyte form of CS microgels was significantly decreased compare with CS microgels, but sustainable and long-term RA delivery was provided for 150 h. Therefore, it can be assumed that the ionic structure of polyelectrolyte CS^−^[R]^+^ microgels could present a milieu that provided more interaction ability with the RA molecules than more negatively charged CS microgels, and sustainable release could be possible, as only 23% of the loaded RA could be delivered because of the high interaction between the drug molecules.

**Figure 7:**
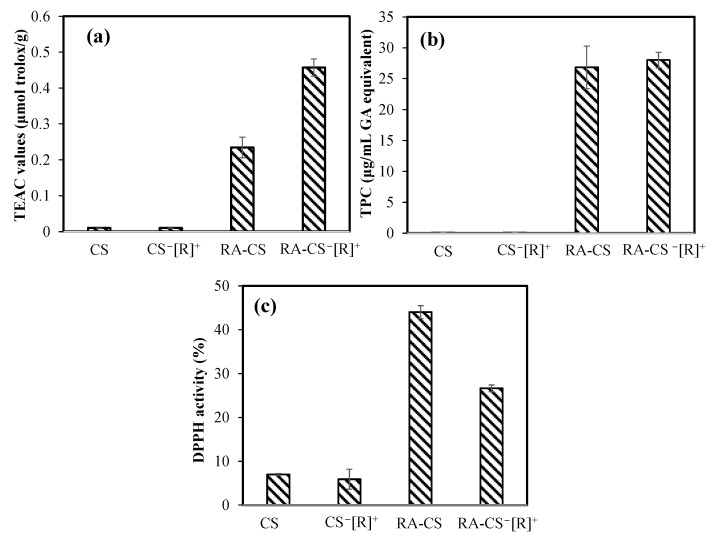
The antioxidant capacities of bare and RA-loaded CS microgels and polyelectrolyte CS^−^[R]^+^ microgels (**a**) via Trolox equivalent antioxidant capacity (TEAC) test, (**b**) gallic acid (GA) equivalent total phenol content (TPC) at 1000 μg/mL concentrations of microgels, and (**c**) DPPH radical-scavenging assay for 3.33 mg/mL concentrations of the microgels.

The antioxidant activities of bare and RA-loaded CS microgels and CS^−^[R]^+^ microgels were investigated by ABTS scavenging, FC, and DPPH scavenging assays, as represented in Figure 7a, Figure 7b and Figure 7c, respectively.

In the ABTS radical-scavenging test, no antioxidant effect was found for bare microgels, but 0.23 ± 0.03 and 0.45 ± 0.02 μmol/g Trolox equivalent antioxidant capacity (TEAC) values were measured for RA-loaded CS microgels and CS^−^[R]^+^ microgels, respectively. As a control, the TEAC value of only RA was determined as 10.32 ± 1.01 μmol/g. In the other antioxidant test, the total phenol contents (TPC) of RA-loaded CS microgels and CS^−^[R]^+^ microgels at 1000 μg/mL concentrations demonstrated an antioxidant capacity of 26.8 ± 3.4 and a 28.0 ± 1.3 μg/mL gallic acid equivalent, respectively. RA at the same concentration had a 556 ± 35 μg/mL gallic acid equivalent TPC value. These results confirm that RA-loaded CS^−^[R]^+^ microgels showed great antioxidant ability, with almost 20-fold lesser antioxidant capacity of sole RA. In the DPPH scavenging activity test, the inhibition percentages of 3.33 mg/mL concentrations of bare CS microgels and CS^−^[R]^+^ microgels were measured as 7.0 ± 0.2 and 5.9 ± 2.2%, respectively. On the other hand, the same amounts of RA-loaded CS microgels and CS^−^[R]^+^ microgels showed higher scavenging ability, with 44.0 ± 1.5 and 26.6 ± 0.7% DPPH inhibition activity, respectively, in about 30 min. The IC_50_ value of RA, which was defined as the concentration of 50% inhibition of the DPPH radical, was reported as 1.3 ± 0.1 μg/mL [42]. According to our results, the highest antioxidant ability in the DPPH assay was determined for CS-RA microgels, with almost 3.33 mg/mL of IC_50_ value.

## 4. Conclusions

Spherical and non-porous CS microgels in the size range of 5 μm to a few hundred nanometers were treated with aqueous ammonia solution to prepare CS^−^[NH_4_]^+^ microgels as polyelectrolyte particles. The zeta potential values of these polyelectrolyte CS^−^[R]^+^ microgels were slightly changed to lesser negative values in the pH range of 5–11 because of the presence of cationic [NH_4_]^+^ groups in the microgel polymeric network. The FT-IR peaks at 3250 and 1414 cm^−1^ assigned to -NH_4_^+^ groups endorsed the polyelectrolyte nature of the CS^−^[R]^+^ microgels. Additionally, CS^−^[R]^+^ microgels demonstrated lesser thermal stability than CS microgels. The CS-based microgels showed a highly biocompatible nature according to a cytotoxicity analysis on L929 fibroblast cells. Moreover, CS^−^[NH_4_]^+^ microgels could be utilized as an active agent carrier, e.g., RA with a 32.4 ± 5.1 μg/mg RA-loading capacity, and could provide sustainable and long-term RA delivery up to 150 h in PBS. Although RA-loaded CS^−^[NH_4_]^+^ microgels exhibited significant antioxidant ability, almost 20-fold lower than that of pure RA, they could be used as antioxidant-carrying particles for some wound-dressing applications, as well as food-packing materials.

## Data Availability

The data presented in this study are available on request from the corresponding author.
